# Machine learning approach for Migraine Aura Complexity Score prediction based on magnetic resonance imaging data

**DOI:** 10.1186/s10194-023-01704-z

**Published:** 2023-12-18

**Authors:** Katarina Mitrović, Andrej M. Savić, Aleksandra Radojičić, Marko Daković, Igor Petrušić

**Affiliations:** 1 Department of Information Technologies, Faculty of Technical Sciences Čačak, University of Kragujevac, 65 Svetog Save, Čačak, 32000 Serbia; 2 Science and Research Centre, University of Belgrade - School of Electrical Engineering, University of Belgrade, 73 Bulevar kralja Aleksandra, Belgrade, 11000 Serbia; 3 Headache Center, Neurology Clinic, University Clinical Centre of Serbia, 6 dr Subotića starijeg, Belgrade, 11000 Serbia; 4 Faculty of Medicine, University of Belgrade, 8 dr Subotića starijeg, Belgrade, 11000 Serbia; 5 Laboratory for Advanced Analysis of Neuroimages, Faculty of Physical Chemistry, University of Belgrade, 12-16 Studentski trg, Belgrade, 11000 Serbia

**Keywords:** Artificial intelligence, Support vector machine, Machine learning, Magnetic resonance imaging, Migraine with Aura, Prediction, regression

## Abstract

**Background:**

Previous studies have developed the Migraine Aura Complexity Score (MACS) system. MACS shows great potential in studying the complexity of migraine with aura (MwA) pathophysiology especially when implemented in neuroimaging studies. The use of sophisticated machine learning (ML) algorithms, together with deep profiling of MwA, could bring new knowledge in this field. We aimed to test several ML algorithms to study the potential of structural cortical features for predicting the MACS and therefore gain a better insight into MwA pathophysiology.

**Methods:**

The data set used in this research consists of 340 MRI features collected from 40 MwA patients. Average MACS score was obtained for each subject. Feature selection for ML models was performed using several approaches, including a correlation test and a wrapper feature selection methodology. Regression was performed with the Support Vector Machine (SVM), Linear Regression, and Radial Basis Function network.

**Results:**

SVM achieved a 0.89 coefficient of determination score with a wrapper feature selection. The results suggest a set of cortical features, located mostly in the parietal and temporal lobes, that show changes in MwA patients depending on aura complexity.

**Conclusions:**

The SVM algorithm demonstrated the best potential in average MACS prediction when using a wrapper feature selection methodology. The proposed method achieved promising results in determining MwA complexity, which can provide a basis for future MwA studies and the development of MwA diagnosis and treatment.

## Background

Migraine with aura (MwA) is a type of migraine that can be manifested very heterogeneously [1]. It is characterized by the aura phase, defined as the complex of fully reversible neurological symptoms, such as visual, somatosensory, speech, motor, brainstem, and/or retinal, that can precede or follow the headache phase [2]. The most common subtype of MwA is migraine with a typical aura, which is present in almost one-third of patients who suffer from migraine [3].

MwA is also characterized by an intra-variability in symptoms that are manifested in different attacks of the same patient [4, 5]. The variability of symptoms during an attack is most often related to the duration, quality, and degree of involvement of aura symptoms [6]. To stratify MwA patients and investigate the correlation between MwA complexity and changes in the cortex structure, a system for assessment of the quality and quantity of MwA attack symptoms in individual patients was introduced [7]. This system for quantifying MwA complexity is based on measuring Migraine Aura Complexity Score (MACS) [7, 8]. MACS reflects the presence and quality of visual, somatosensory, dysphasic, and other higher cortical symptoms, and it is developed to assess the aura complexity and stratification of MwA subtypes, which can significantly improve the investigation of MwA pathophysiology and point to new targets and solutions for individual and precision medicine treatment of MwA patients [7]. MACS is measured for each attack individually and provides insight into attack complexity. By averaging multiple scores in one patient, a quantitative value of average MwA complexity for a selected patient can be obtained.

Magnetic resonance imaging (MRI) data was previously used in a limited number of studies to explore the connection between MwA complexity and changes in the cortex [6–13]. MwA is commonly categorized into simple MwA (MwA-visual) and complex MwA (MwA-visual plus) based on the occurring symptoms, where MwA-visual includes visual symptoms only, whereas the presence of somatosensory and/or dysphasic symptoms in addition to the visual ones indicates MwA-visual plus [6, 9, 10, 12–14]. In the MwA-visual plus group of patients, it has been shown that there is a significantly reduced surface area and volume of the left rostral middle frontal cortex, as well as increased left temporal pole sulcal depth in comparison to MwA-visual, which reinforces the statement that structural measures of cortical regions can be used as potential biomarkers of MwA subtypes of different complexity [6]. Other results argue that MwA-visual and MwA-visual plus can be distinguished based on their cortical thickness, surface area, volume, mean Gaussian curvature, and folding index of particular regions and suggest further research [13]. Moreover, a recent study revealed that MwA is associated with thinning in various cortical regions, and the clinical diversity of aura symptoms is mirrored by contrasting thickness alterations in regions responsible for high-level visual processing, sensorimotor function, and language processing [12]. Furthermore, the previous studies which investigated differences between MwA patients who have MwA-visual and those who have MwA-visual plus symptoms using functional MRI found significant alterations in multiple brain regions such as the visual cortex, lingual gyrus, anterior insula, and sensorimotor cortex [9–11], but did not perform further investigation using structural MRI.

Previous reports show evidence of a relation between MACS and the structural MRI data [7, 8]. More specifically, a thicker left primary visual cortex in the MwA groups with higher MACS was found, as well as a thicker cortex in several visual and somatosensory cortical regions of patients with high MACS relative to the patients with low MACS values [8]. Furthermore, the MACS demonstrated a positive correlation with the cortical thickness of multiple brain regions, where the left and right lateral occipital, right cuneus, right precuneus, left postcentral, and left and right superior parietal cortices showed the greatest significance [7]. This work extends this investigation with focusing on the average MACS score and studying its correlation with the comprehensive set of cortical features derived from structural MRI data.

The main goal of this study is to test the feasibility of predicting the average MACS from the structural MRI data using advanced ML algorithms and feature selection methods. Moreover, this study aims to offer scientific directions of the exploration of novel machine learning (ML) approaches, combined with comprehensive structural MRI data of the cerebral cortex, for investigating MwA complexity.

## Methods

### Participants

MwA patients included in the study were from the cohort of patients who were enrolled in a previous migraine neuroimaging study [13]. Diagnosis of episodic migraine with typical aura was established based on the third edition of the International Classification of Headache Disorders (ICHD-3) criteria [2]. This research was conducted in accordance with the ethical standards of the institutional and/or national research committee and with the 1964 Declaration of Helsinki and its later amendments or comparable ethical standards. In addition, all procedures related to the data set preparation were approved by the Review Board of the Neurology Clinic.

MwA patients had to meet the following criteria to be included in the study: 1) written consent of participation in the study, 2) 21-55 years of age, 3) episodic migraine with typical aura present for five years or more, 4) two or more MwA attacks per year, 5) have never used migraine preventive therapy, and 6) right-hand side of body predominance (to avoid possible differences in brain regions).

In case the presence of other headache types, neurological, cardiovascular, or metabolic disorders were identified in medical history or during a physical examination, the participant was excluded from the study. Occasional migraine without aura or tension headaches were allowed. Claustrophobia or incapacity to undergo an MRI examination implied an inability to participate in this study. Subjects with structural abnormalities recorded on MRI were also withdrawn from the study. Also, MwA patients were scanned during the interictal stage of the migraine cycle.

In addition to MRI data, the average MACS values for each participant were acquired. MACS score is determined based on the questionnaire that is fulfilled after every MwA attack [7, 8]. The questionnaire consists of questions related to the symptoms that the patient may have experienced during the MwA attack. In particular, patients who had experienced visual disturbances also reported the level of involvement of the visual field, while patients who had experienced somatosensory symptoms also reported the number of body regions that were involved. Also, patients have reported if they experienced some kind of higher cortical dysfunctions, including higher cortical disturbances of visual (micropsia, macropsia, dysmorphia, fractured vision, and prosopagnosia) and somatosensory symptoms (astereognosis, dyspraxia, and unawareness of one’s own body parts), as well as dysphasic and memory disturbances. More detailed information about the questionnaire can be found elsewhere [7, 8]. Additionally, to create a more precise average score, the average MACS of a minimum of 6 MwA attacks was calculated and used as a final score. The range of the average MACS can be from 0 to 9, where a 0 value indicates the presence of MwA with mild forms of aura and higher values of MACS indicate a more complex aura.

### MRI data acquisition and post-processing

The MRI examination was performed on a 3 T Scanner (MAGNETOM Skyra, Siemens, Erlangen, Germany). Protocol for MRI examination was: 1) 3D T1 (repetition time (TR) = 2300 ms, echo time (TE) = 2.98 ms, flip angle (FA) = 9$^{\circ }$, 130 slices with voxel size 1 $\times$ 1 $\times$ 1 mm$^3$, acquisition matrix 512 $\times$ 512, field of view (FOV) = 256 $\times$ 256 mm$^2$), 2) 3D FLAIR (TR = 5000 ms, TE = 398 ms, inversion time = 1800 ms, FA = 120$^{\circ }$, acquisition matrix 256 $\times$ 256, FOV = 256 $\times$ 256 mm$^2$), and 3) T2 weighted spin echo (T2W) in an axial plane (TR= 4800 ms, TE = 92 ms, FA = 90$^{\circ }$, acquisition matrix 384 $\times$ 265, FOV= 256 $\times$ 256 mm$^2$, slice thickness = 5 mm). T2W images were only used to exclude the presence of brain lesions.

Freesurfer (v 6.0) analysis was performed on an HP DL850 server (Intel Xeon 3.2 MHz, eight cores, 16 GB RAM) using a recon-all script, combining 3D T1 and FLAIR images, for automatic cortical reconstruction and segmentation of brain structures. The average run time (with the parallelization option used) was six hours. Details about Freesurfer and its routines can be found in other studies [15]. Cortical parcellation was done according to the Desikan-Killiany Atlas [16].

Based on MRI images, data were obtained for the left and right hemispheres of the brain. For each hemisphere, data was collected for 34 regions of the cortex, namely: banks of the superior temporal sulcus, caudal anterior cingulate, caudal middle frontal, cuneus, entorhinal, fusiform, inferior parietal, inferior temporal, isthmus cingulate, lateral occipital, lateral orbitofrontal, lingual, medial orbitofrontal, middle temporal, parahippocampal, paracentral, pars opercularis, pars orbitalis, pars triangularis, pericalcarine, postcentral, posterior cingulate, precentral, precuneus, rostral anterior cingulate, rostral middle frontal, superior frontal, superior parietal, superior temporal, supramarginal, frontal pole, temporal pole, transverse temporal, and insula region. For each region, thickness, surface area, volume, mean Gaussian curvature and folding index were measured. The data set consists of 340 input features of numerical data type. The dimension of the dataset can be represented as follows:

1 $$\begin{aligned} h \times r \times m = 2 \times 34 \times 5 = 340 \end{aligned}$$

where h represents brain hemispheres, r represents 34 regions, and m refers to 5 observed measures.

### Statistical analysis

The sample size was based on the available data and previous literature [7, 13]. Furthermore, according to the recommendation for clinical research [17], the total sample size required to determine whether a correlation coefficient differs from zero is 29 participants, when $\alpha$ (two-tailed) is set to 0.05, $\beta$ (type II error rate) is set to 0.2 and the expected correlation coefficient is set to 0.5. Accordingly, 40 MwA patients were included in this study. Based on the assumption that the complexity of MwA is associated with changes in some regions of the cortex, a correlation analysis between each feature from the data set and the average MACS was performed. The correlation was performed 340 times for each input feature individually, where the first variable was a feature from the input set, and the second variable was the average MACS. Given that the average MACS variable is not normally distributed, and it participates in measuring all correlation coefficients, the assumption of bivariate normality cannot be justified. Therefore, this statistical analysis was carried out using Spearman’s rank correlation coefficient [18, 19]. The threshold for statistical significance (*p*-value) was 0.05.

### Machine learning

The feature selection and ML model training were performed in the Waikato Environment for Knowledge Analysis (Weka) software.

The majority of ML algorithms operate based on the assumption that there are more samples than predictors in the data set. In cases where the number of inputs exceeds the number of samples, the problem of dimensionality of the data set may occur. This can result in a high variance and overfitting, which can distort the prediction results [20]. The data set for ML algorithm training in this study includes 40 samples and 340 input features ($p=340$, $N=40$), which indicates a dimensionality problem. Therefore, before training the regression ML algorithm, the feature selection should be performed, thereby reducing the dimensionality of the data set.

In this study, two approaches were used for feature selection: a correlation-based selection and a wrapper method-based selection. The first method is based on the Fast correlation-based filter solution which implies calculating the correlation between continuous variables and output, deriving a statistically significant subset of features, and then removing redundant features based on feature-feature correlation [21]. The feature subsets derived with Spearman’s rank statistical analysis when $p < 0.05$, as well as when $p < 0.01$, are used for the correlation-based approach. After calculating the correlation coefficient between features, the pairs of features that correlated with a coefficient greater than or equal to 0.85 were determined. The redundant features are eliminated based on their correlation with MACS, where the feature correlating with MACS at a lower significance level was retained in each pair. The features remaining after the process of elimination were used as input for ML algorithm training.

The second method for feature selection used the wrapper method which performs an extensive search of the feature space and returns a subset of features that achieves the best results using a learning scheme [22]. The estimation of the correlation coefficient is determined using 5-fold cross-validation. The search method used for this feature selection is the best first algorithm. It performs a forward search of the space of feature subsets where the starting point includes the empty set of features.

The ML models implemented for prediction of average MACS are the Support Vector Machine (SVM) algorithm for regression or Support Vector Regression (SVR), Linear Regression (LR), and Radial Basis Function (RBF) network.

The basic idea of SVR is finding a function that has a low deviation from the output values while maintaining the shape as flat as possible to preserve the generalization ability [23]. SVR model can be written as:

2 $$\begin{aligned} f(x)=\sum \limits _{i=1}^{N} \left( \alpha _{i}-\alpha _{i}^{*}\right) \langle x_i,x\rangle +b \end{aligned}$$

where x represents the input data, i is the data index (i$\in$1,...,N), $\alpha$ is Lagrange multiplier, b is the bias, and $\langle x_i,x\rangle$ is the dot product of its elements [23]. The optimal solution of SVR has to satisfy Karush-Kuhn-Tucker conditions:

3 $$\begin{aligned} \alpha _i(\varepsilon +\xi _i-y_i+\langle w,x_i\rangle +b)=0 \end{aligned}$$

4 $$\begin{aligned} \alpha _i^*(\varepsilon +\xi _i^*+y_i-\langle w,x_i\rangle -b)=0 \end{aligned}$$

as well as conditions:

5 $$\begin{aligned} (C-\alpha _i )\xi _i=0 \end{aligned}$$

6 $$\begin{aligned} \left(C-\alpha _i^*\right)\xi _i^*=0 \end{aligned}$$

where y is the output data, w is a vector normal to the hyperplane, $\varepsilon$ is a threshold to which the deviations are tolerated, C is the complexity parameter that determines the trade-off between the flatness of the model and the error tolerance, and $\xi$ and $\xi ^*$ are slack variables that make the optimization problem feasible [23].

For solving regression problems with SVM, an iterative model called Sequential Minimal Optimization (SMO) is used. This research employs a modified SMO algorithm where two threshold parameters are being maintained [24]:

7 $$\begin{aligned} \tilde{F}_{i_{low}}=b_{low}=max \{\ \tilde{F}_i : i\in I_0\cup I_1\cup I_2 \} \end{aligned}$$

8 $$\begin{aligned} \bar{F}_{i_{up}}=b_{up}=min \{\ \bar{F}_i : i\in I_0\cup I_1\cup I_3 \} \end{aligned}$$

The following formulas represent $\tilde{F}_i$ and $\bar{F}_i$:

9 $$\begin{aligned} \tilde{F}_i = \left\{ \begin{array}{cl} y_i-\langle w,x_i \rangle +\epsilon &{} : \ i\in I_{0b}\cup I_2 \\ y_i-\langle w,x_i \rangle -\epsilon &{} : \ i\in I_{0a}\cup I_1 \end{array} \right. \end{aligned}$$

10 $$\begin{aligned} \bar{F}_i = \left\{ \begin{array}{cl} y_i-\langle w,x_i \rangle +\epsilon &{} : \ i\in I_{0b}\cup I_1 \\ y_i-\langle w,x_i \rangle -\epsilon &{} : \ i\in I_{0a}\cup I_3 \end{array} \right. \end{aligned}$$

Index sets for $\alpha$ are defined as follows:

11 $$\begin{aligned} I_{0a} = \left\{ i : \ 0< \alpha _i< C \right\} \end{aligned}$$

12 $$\begin{aligned} I_{0b} = \left\{ i : \ 0< \alpha _i^*< C \right\} \end{aligned}$$

13 $$\begin{aligned} I_1 = \left\{ i : \ \alpha _i=0, \ \alpha _i^*=0 \right\} \end{aligned}$$

14 $$\begin{aligned} I_2 = \left\{ i : \ \alpha _i=0, \ \alpha _i^*=C \right\} \end{aligned}$$

15 $$\begin{aligned} I_3 = \left\{ i : \ \alpha _i=C, \ \alpha _i^*=0 \right\} \end{aligned}$$

The following condition is used for optimality checking:

16 $$\begin{aligned} b_{low}\le b_{up}+2\tau \end{aligned}$$

where $\tau$ is a positive tolerance parameter [24].

The kernel function used with SVM in this research is linear kernel, which can be mathematically represented as follows [25]:

17 $$\begin{aligned} K(x_i,x_j )=x_i x_j \end{aligned}$$

where $x_i$ and $x_j$ are the samples of data. The complexity parameter C was set to 1, whereas the epsilon parameter of the epsilon insensitive loss function and the tolerance were equal to 0.001. Before applying the algorithm, the normalization of data was performed.

The basic LR can be mathematically represented as follows:

18 $$\begin{aligned} Y=X\beta +\varepsilon \end{aligned}$$

where Y is the output vector, X is the input matrix, $\beta$ is the unknown parameter vector, and $\varepsilon$ is the vector of errors [26]. The model selection is based on the M5 model tree and Akaike information criterion. In each iteration, the feature with the lowest standardized coefficient is eliminated until a stagnation in the decrease of the estimated error is noted.

RBF network is a three-layer feed-forward neural network that uses Gaussian RBF as an activation function and it can be computed as follows:

19 $$\begin{aligned} h(x)= e^{-\frac{\left\| x-c\right\| ^2}{\sigma ^2}} \end{aligned}$$

where c is the center and $\sigma ^2$ is the variance [27]. The center is a fixed point that represents the central point of each node, whose initial value is determined using the k-means clustering algorithm. The output calculation is based on the Euclidean distance between the data point and a set of centers. The output calculation is based on the activation functions of hidden units (18) and the function weights:

20 $$\begin{aligned} f(x)=\sum \limits _{i=1}^{N} w_i h_i (x) \end{aligned}$$

The penalized squared error is minimized using the quasi-Newton method based on the Broyden-Fletcher-Goldfarb-Shanno (BFGS) updates. The ridge hyperparameter of the error function that indicates the weight penalty and controls overfitting is set to 0.01. Another hyperparameter of this algorithm is the number of RBFs, which was set to 4. Before applying the algorithm, the normalization of data was performed.

The quality metrics used to determine the success of the prediction are coefficient of determination or R2 score (R2), mean absolute error (MAE), and root mean squared error (RMSE). R2 is based on calculating the sum of squares of the residual errors and the total sum of the errors:

21 $$\begin{aligned} R^2=1-\frac{\sum _{i=1}^{n}(y_i-\hat{y}_i )^2}{\sum _{i=1}^{n}(y_i-\bar{y})^2} \end{aligned}$$

where n is the sample size, y is the real output value, $\hat{y}$ is the predicted value, and $\bar{y}$ is the mean value of y [28]. The value of R2 ranges between 0 and 1 where higher values indicate better performance of the model. MAE can be defined as follows:

22 $$\begin{aligned} MAE= \frac{1}{n} \sum _{i=1}^{n}|y_i-\hat{y}_i| \end{aligned}$$

whereas RMSE is defined as:

23 $$\begin{aligned} RMSE=\sqrt{\frac{1}{n} \sum \limits _{i=1}^{n}\left(y_i-\hat{y}_i\right)^2} \end{aligned}$$

where n is the sample size, y is the real output value, and $\hat{y}$ is the predicted value [29].

The quality of the prediction was evaluated using the 10-fold cross-validation method.

## Results

This study included data from 40 MwA patients. The main demographic data and aura features of participants including gender, age, average MACS score, duration of the aura, and attack frequency are presented in Table 1.

**Table 1:** Characteristics of participants

**Variable**	**MwA** ^**a**^ **(*n*** **= 40)**
Female, number of participants (%)	27 (67.50 %)
Age, mean ± SD ^b^ (range)	36.20 ± 8.90 (20.00 - 55.00)
MACS ^c^, mean ± SD (range)	2.86 ± 2.40 (0.00 - 7.50)
Aura duration (minutes), mean ± SD (range)	36.25 ± 17.05 (10.00 - 90.00)
Attack frequency per year, mean ± SD (range)	6.20 ± 6.75 (1.00 - 30.00)

^a^ *MwA* migraine with aura, ^b^ *SD* standard deviation, ^c^ *MACS* migraine aura complexity score

The initial data set in this study contained 340 input features obtained by MRI scanning, including thickness, surface area, volume, mean Gaussian curvature, and folding index of cortical regions of both brain hemispheres. The feature-MACS correlation established 26 features with a significant relationship with the average MACS ($p < 0.05$). These features, as well as their correlations to the average MACS and *p*-values, are shown in Table 2.

**Table 2:** Features with significant correlation with average MACS

**Feature**	**Correlation**	***p*-value**
Left parahippocampal gyrus mean Gaussian curvature	-0.4763	0.0019
Left transverse temporal gyrus mean Gaussian curvature	0.4748	0.0020
Left transverse temporal gyrus thickness	0.4693	0.0023
Left pars opercularis thickness	0.4674	0.0024
Left lingual gyrus surface area	-0.4191	0.0071
Right transverse temporal gyrus mean Gaussian curvature	0.4072	0.0091
Left caudal middle frontal gyrus surface area	-0.3964	0.0113
Left entorhinal gyrus volume	-0.3737	0.0175
Right entorhinal gyrus volume	-0.3736	0.0176
Right lingual gyrus surface area	-0.3711	0.0184
Left precuneus gyrus surface area	-0.3572	0.0237
Left pars opercularis surface area	-0.3545	0.0248
Right caudal middle frontal gyrus surface area	-0.3510	0.0264
Right transverse temporal gyrus thickness	0.3480	0.0278
Left caudal middle frontal gyrus volume	-0.3461	0.0287
Left isthmus cingulate surface area	-0.3367	0.0336
Left pericalcarine thickness	0.3286	0.0384
Left caudal anterior cingulate mean Gaussian curvature	0.3284	0.0386
Right entorhinal gyrus surface area	-0.3276	0.0391
Right pericalcarine mean Gaussian curvature	-0.3214	0.0431
Left medial orbitofrontal gyrus folding index	-0.3208	0.0436
Left pars triangularis thickness	0.3186	0.0451
Right caudal middle frontal gyrus folding index	-0.3175	0.0459
Right paracentral gyrus mean Gaussian curvature	0.3167	0.0465
Left lingual gyrus volume	-0.3159	0.0470
Right lingual gyrus volume	-0.3141	0.0484

**Fig. 1:**
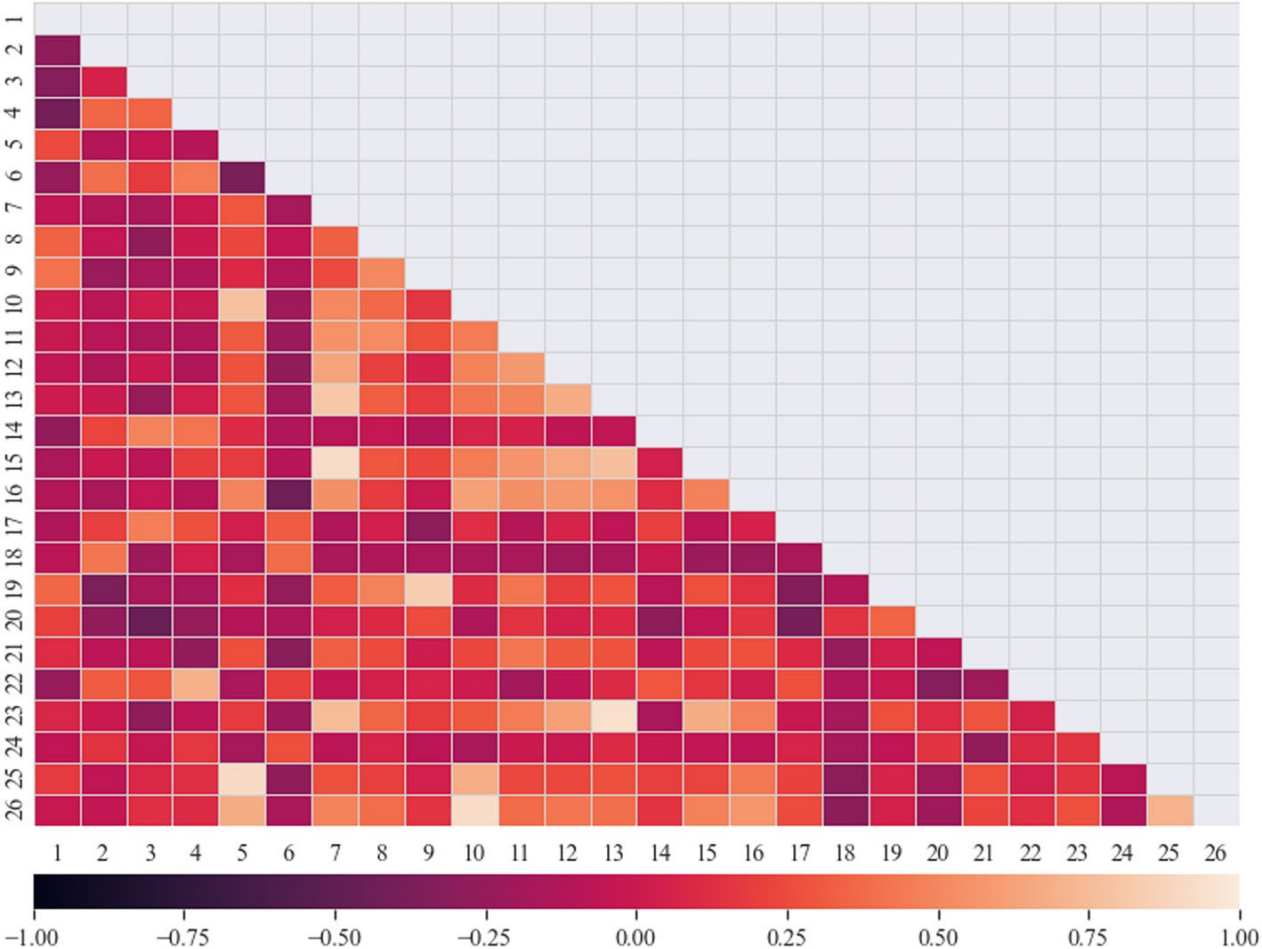
Correlation analysis of features that significantly correlate with MACS (*p *< 0.05). The correlation-based feature selection includes the elimination of features that have a correlation greater than ±0.85. Correlation values range from -1 to 1 as shown in the heatmap color spectrum. The purple color symbolises a negative correlation, the middle shades indicate a low correlation, whereas the orange section indicates a positive correlation. The color intensity shows the correlation strength. (1 - Left parahippocampal gyrus mean Gaussian curvature; 2 - Left transverse temporal gyrus mean Gaussian curvature; 3 - Left transverse temporal gyrus thickness; 4 - Left pars opercularis thickness; 5 - Left lingual gyrus surface area; 6 - Right transverse temporal gyrus mean Gaussian curvature; 7 - Left caudal middle frontal gyrus surface area; 8 - Left entorhinal gyrus volume; 9 - Right entorhinal gyrus volume; 10 - Right lingual gyrus surface area; 11 - Left precuneus gyrus surface area; 12 - Left pars opercularis surface area; 13 - Right caudal middle frontal gyrus surface area; 14 - Right transverse temporal gyrus thickness; 15 - Left caudal middle frontal gyrus volume; 16 - Left isthmus cingulate surface area; 17 - Left pericalcarine thickness; 18 - Left caudal anterior cingulate mean Gaussian curvature; 19 - Right entorhinal gyrus surface area; 20 - Right pericalcarine mean Gaussian curvature; 21 - Left medial orbitofrontal gyrus folding index; 22 - Left pars triangularis thickness; 23 - Right caudal middle frontal gyrus folding index; 24 - Right paracentral gyrus mean Gaussian curvature; 25 - Left lingual gyrus volume; 26 - Right lingual gyrus volume)

Further, a correlation analysis that determined correlation coefficients between these features was conducted, where four pairs of highly correlated features were detected (Fig. 1). Within each pair, the feature correlating with MACS at a lower significance level was retained. The eliminated features are left and right lingual volume, left caudal middle frontal gray volume, and right caudal middle frontal folding index.

**Fig. 2:**
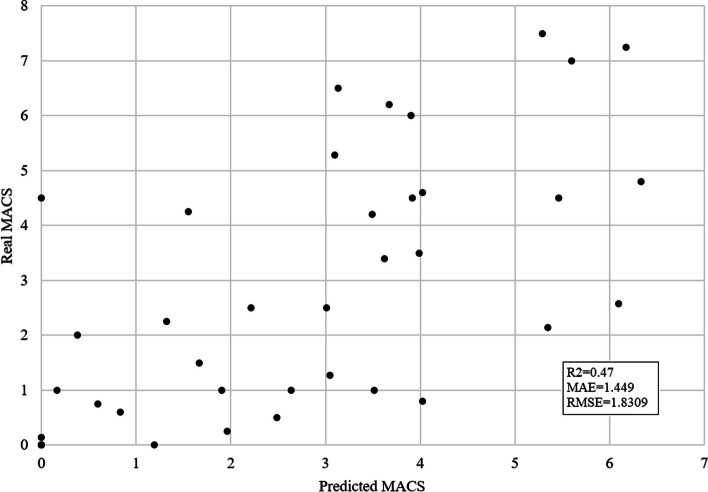
Predicted vs. real average MACS for SVM algorithm and correlation-based feature selection (*p *< 0.05). The x-axis shows predicted average MACS and the y-axis real average MACS scores. The prediction is performed using the SVM algorithm and features that correlate with average MACS with a 0.05 significance level. Each black dot represents one subject. This model achieved R2 = 0.47, MAE = 1.449, and RMSE = 1.8309

The final data set which was used for ML model training when correlation-based feature selection was performed contained 22 features. The SVM regressor outperformed other algorithms with the following results: R2 = 0.47, MAE = 1.449, and RMSE = 1.8309. Figure 2 shows the difference between the real and predicted average MACS scores using this model.

**Fig. 3:**
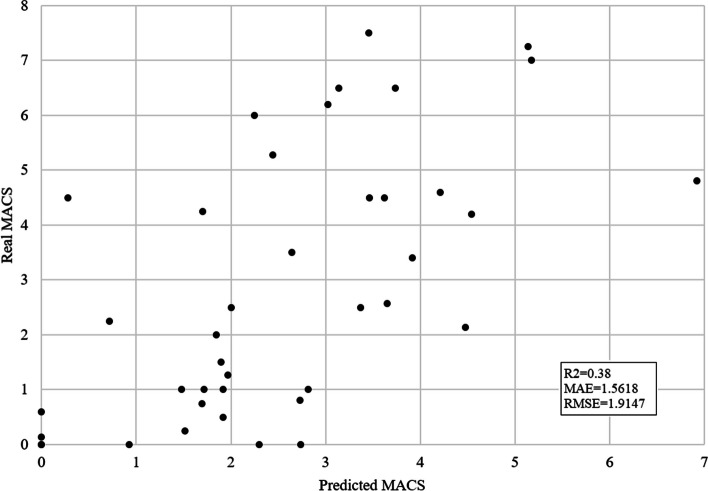
Predicted vs. real average MACS for SVM algorithm and correlation-based feature selection (*p *< 0.01). The x-axis shows predicted average MACS and the y-axis real average MACS scores. The prediction is performed using the SVM algorithm and features that correlate with average MACS with a 0.01 significance level. Each black dot represents one subject. This model achieved R2 = 0.38, MAE = 1.5618, and RMSE = 1.9147

The subset of data that included 6 features that were the result of correlation analysis with a significance level of 0.01 was also evaluated for the prediction of average MACS. Although the SVM regressor exceeded other algorithms, the results showed a notable decline: R2 = 0.38, MAE = 1.5618, and RMSE = 1.9147. Figure 3 shows the difference between the real and predicted average MACS scores using this model.

The wrapper method for feature selection was applied to help reveal less obvious subsets of features that undergo changes with the increased complexity of MwA and thus collectively contribute to better prediction of average MACS scores. The data subset derived using wrapper feature selection contained the following 18 input features:

- Left caudal middle frontal surface area;
- Left inferior parietal volume;
- Left inferior temporal thickness;
- Left isthmus cingulate volume;
- Left lingual surface area;
- Left parahippocampal mean Gaussian curvature;
- Left pericalcarine mean Gaussian curvature;
- Left postcentral surface area;
- Left transverse temporal thickness;
- Left transverse temporal mean Gaussian curvature;
- Right fusiform surface area;
- Right isthmus cingulate folding index;
- Right medial orbitofrontal thickness;
- Right middle temporal folding index;
- Right parahippocampal folding index;
- Right pars orbitalis folding index;
- Right postcentral surface area;
- Right postcentral volume.

This selection of features provided a subset that showed great potential in the prediction of average MACS. Table 3 shows SVM, LR, and RBF network results. The model training was carried out using 10-fold cross-validation.

**Table 3:** The results of machine learning algorithms with wrapper feature selection

**Algorithm**	**R2** ^**a**^	**MAE** ^**b**^	**RMSE** ^**c**^
SVM ^d^	0.8934	0.5229	0.7971
LR ^e^	0.8046	0.8710	1.0827
RBF ^f^ network	0.7309	0.9586	1.2321

^a^ *R2* coefficient of determination, ^b^ *MAE* mean absolute error, ^c^ *RMSE* root mean squared error, ^d^ *SVM* support vector machine, ^e^ *LR* linear regression, ^f^ *RBF* radial basis function

**Fig. 4:**
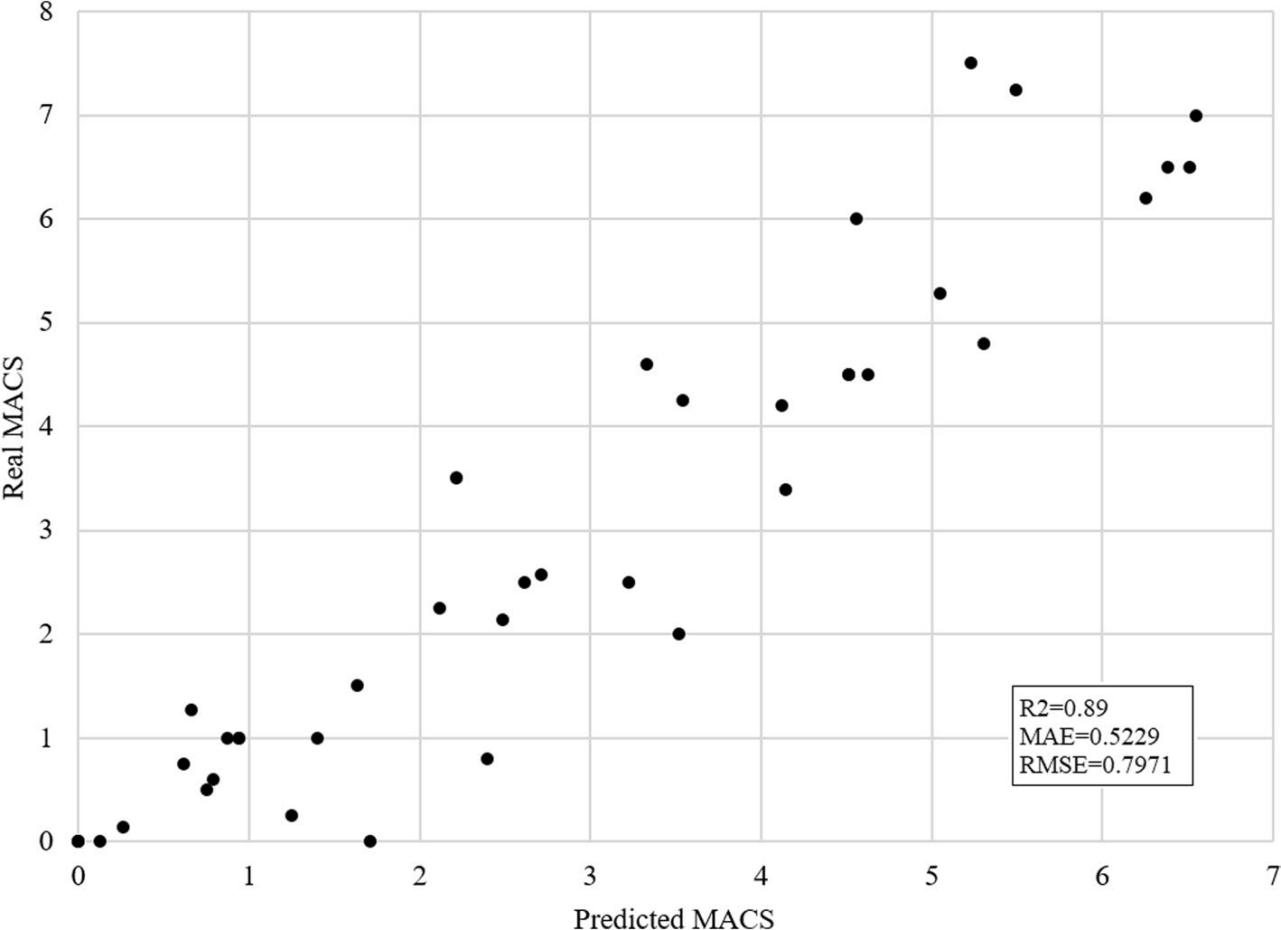
Predicted vs. real average MACS for SVM algorithm and wrapper feature selection subset. The x-axis shows predicted average MACS and the y-axis real average MACS scores. The prediction is performed using the SVM algorithm and features that were derived with the wrapper feature selection method. Each black dot represents one subject. This model achieved R2 = 0.8046, MAE = 0.871, and RMSE = 1.0827

RBF neural network achieved R2 = 0.7309, MAE = 0.9586, and RMSE = 1.2321. LR algorithm performed normalization and iterative selection of data. This model for average MACS prediction achieved R2 = 0.8046, MAE = 0.871, and RMSE = 1.0827. SVM results include R2 = 0.89, MAE = 0.5229, and RMSE = 0.7971, which notably outperformed other models. Figure 4 presents the predicted vs. real MACS values using wrapper feature selection and SVM regression.

## Discussion

The focus of this study is to find brain regions that show alterations with different aura complexity. Statistical analysis and ML techniques were implemented to identify these regions with the goal of finding markers to aid the diagnosis of MwA and the level of its complexity. Correlation analysis of the input features and average MACS was conducted, resulting in a subset of features that show significant alterations with different average MACS scores. Further, this study aimed to explore the potential of ML algorithms in the average MACS score prediction to provide a direction for future research in this domain. Different feature selection methods were tested and the derived data subsets were used as a basis for implementation of the ML algorithms which led to identifying important predictors of the MwA complexity in patients.

Several studies have been devoted to finding reliable markers to identify the presence of MwA and the approaches and results are varied [6–8, 10–13]. This paper represents efforts to strengthen and expand the results in this area. The dataset used in this research included 340 cortical features, which were the input for the statistical analysis along with the average MACS score. This study extends previous research based on MACS values. This score is a relatively new concept whose potential is being explored and additional research is needed in this area. Correlation analysis between features and average MACS score identified 26 significant features that strongly correlated with average MACS ($p < 0.05$). Most prominent features include parahippocampal mean Gaussian curvature, transverse temporal mean Gaussian curvature, transverse temporal thickness, pars opercularis thickness, and lingual surface area of the left hemisphere, and transverse temporal mean Gaussian curvature of the right hemisphere ($p < 0.01$). Changes in the structure and function of the parahippocampal gyrus were observed in people with migraine in earlier studies [30, 31], and our results indicate that the morphology of this brain region plays a significant role in the manifestation of the migraine aura and its degree of complexity. Furthermore, our results pointed transverse temporal gyrus as a predominant cortical structure in these findings, therefore its role should be further explored in aura complexity prediction. Other studies found significant changes in transverse temporal gyrus in migraine patients when compared to healthy controls (HCs), as well as across age groups of migraine patients [32, 33], but its role in the aura complexity remains unclear. Also, our findings identify left pars opercularis thickness as one of the most prominent markers for MACS complexity prediction, which is in agreement with the results of another study where this marker is recognized as important for MwA classification using the Linear Discriminant Analysis (LDA) algorithm with a high rate of accuracy [13]. Further, left and right lingual gyrus surface area and volume are found to be significantly correlated with average MACS, and several other studies also found significant changes in the left and right lingual gyrus in MwA patients [10–12]. These findings might indicate that a part of the cortex centralized in the lingual gyrus is involved in the initiation and/or propagation of MwA [11]. Our results confirm the hypothesis that MwA patients exhibit alterations in visual pathways [10, 11], although these studies are based on resting-state functional MRI which allows the exploration of whole-brain functional connectivity.

With the progress of artificial intelligence, primarily ML models, and their extensive application, the number of works on the topic of migraine subtype prediction is increasing. Several studies that use ML models to classify different migraine patients have been conducted [13, 34, 35]. A study that used MRI data of cortical thickness, surface area, and volume performed a set of migraine classification tasks with a high success rate: migraine vs. HCs (68% accuracy), episodic migraine vs. HCs (67% accuracy), chronic migraine vs. HCs (86% accuracy), and chronic vs. episodic migraine (84% accuracy) [34]. LDA algorithm based on post-processed MRI data obtained 97% classification accuracy of MwA patients vs. HCs and 98% accuracy between MwA-visual and MwA-visual plus [13]. Another study developed a model based on the classification methods, feature-selection techniques, and statistical analyses on functional connectivity measures extracted from electroencephalography (EEG) signal to differentiate between MwA and migraine without aura, which achieved 84.62% accuracy [35]. The prediction of migraine and its subtypes is carried out by applying different ML algorithms and using different types of input data such as functional MRI, structural MRI, EEG, and more, which can be a significant aid in the diagnosis and treatment of this disease. This study combines statistical analysis, ML, and previous knowledge about the MACS score to find predictors of migraine complexity. As the MACS is a relatively new concept, its potential is still being explored in current research.

This study was initiated from the assumption that by extracting individual attributes that significantly correlate with the average MACS score, a set of data that will contribute to the prediction of this score can be obtained. However, the research showed that the individual approach had significantly lower performance than using a wrapper method that discovered a combination of features that can perform a good prediction. This strengthens previous suggestions that MwA might be a neural network disease that causes multiple structural changes in the cerebral cortex [13].

In this paper, multiple ML algorithms were applied to several different inputs derived as a result of different feature selection methods. The most prominent algorithms are SVM, LR, and RBF neural network. For each algorithm, a comprehensive search was performed in the hyperparameter space to find those values that contribute the most to a good prediction. The two data sets are established using correlation-based feature selection where features that correlated with MACS with 0.05 and 0.01 level of significance were taken as input sets. In addition, an extensive wrapper feature selection was implemented and resulted in the discovery of a set of features that combined provide promising results in the area of aura complexity prediction. The SVM model for regression achieved a high R2 score and low error values and demonstrated the ability to predict the average MACS score based on these cortical markers.

In other recent studies, the SVM model showed promising results in migraine prediction [36–38]. One study investigated the ability of an SVM model to differentiate migraine patients from HCs and identified the most predictive brain regions [36]. Multi-kernel SVM achieved 83.67% accuracy when classifying migraine patients without aura and controls based on functional and structural MRI data. Similarly, our study is deploying a feature selection and SVM algorithm, although we focus on MwA complexity and related markers. Another finding demonstrates the potential of SVM as a diagnostic tool for migraine without aura with almost 82% accuracy when applied to functional MRI data [37]. Multiple state-of-the-art ML algorithms including SVM were also used to classify migraine patients and HCs, along with feature selection and dimensionality reduction algorithms to build an optimal feature set by removing redundant features [38]. Another common task that yielded the implementation of the ML approach dealt with the classification of MwA vs. migraine without aura [10, 35]. To the best of our knowledge, this is the first study that focused on MwA complexity prediction based on MACS scores and MRI data of the cerebral cortex.

The main limitation of this research lies in the number of participants. A study that includes data from a greater number of subjects would be crucial for confirming our findings. In addition, it can be noted that most of the previous works are based on research on one modality of data. However, we used multidimensional structural MRI data and advanced ML algorithms to improve knowledge about connections between the level of aura complexity and cortical features. Conducting research on different modalities and their combinations (functional MRI, diffusion MRI, structural MRI) can yield significant results and new knowledge in this field. Further, investigation of an interplay between the symptoms of headache, MACS, and cortical features is needed in future studies to gain new insights in this field.

## Conclusions

The ML model based on the SVM algorithm showed potential in predicting average MACS scores using structural MRI data. Also, our findings show that the utilization of advanced ML algorithms can significantly outperform traditional statistical correlation methods for the prediction of the average MACS using structural MRI data of the cerebral cortex. In addition, the findings of this study suggest that the combination of cortical features that are most correlated with the average MACS does not necessarily serve as a good model for prediction, whereas cortical features derived from advanced ML algorithms yield promising results. Altogether, given that increasing MACS implies more abundant symptomatology during the aura phase it does not come as a surprise that advanced ML algorithms pointed to various cortical features spread out throughout a whole brain network affecting predominately parietal and temporal regions. The results of this paper can provide a basis for future MwA studies and the development of evidence-based diagnosis of MwA subtypes and their treatment.

## Data Availability

The dataset used and analysed during the current study is available from the corresponding author on reasonable request.

## Abbreviations

MwA: Migraine with aura
MACS: Migraine Aura Complexity Score
MRI: Magnetic resonance imaging
MwA-visual: Simple migraine with aura
MwA-visual plus: Complex migraine with aura
ML: Machine learning
ICHD-3: International Classification of Headache Disorders, 3rd edition
TR: Repetition time
TE: Echo time
FA: Flip angle
FOV: Field of view
T2W: T2 wighted spin echo
Weka: Waikato Environment for Knowledge Analysis
SVM: Support Vector Machine
SVR: Support Vector Regression
LR: Linear Regression
RBF: Radial Basis Function
SMO: Sequential Minimal Optimization
BFGS: Broyden-Fletcher-Goldfarb-Shanno
R2: Coefficient of determination
MAE: Mean absolute error
RMSE: Root mean squared error
HCs: Healthy controls
LDA: Linear Discriminant Analysis
EEG: Electroencephalography
